# Analysis of Environmental and Pathogenic Bacteria Attached to Aerosol Particles Size-Separated with a Metal Mesh Device

**DOI:** 10.3390/ijerph19095773

**Published:** 2022-05-09

**Authors:** Xiaobo Yin, Seiji Kamba, Koki Yamamoto, Atsushi Ogura, Ernest Apondi Wandera, Mohammad Monir Shah, Hirokazu Seto, Takashi Kondo, Yoshio Ichinose, Makoto Hasegawa

**Affiliations:** 1 Graduate School of Bioscience, Nagahama Institute of Bio-Science and Technology, 1266 Tamura, Nagahama 526-0829, Japan; b112023@m.nagahama-i-bio.ac.jp (X.Y.); pxg02010@nifty.com (S.K.); koki.yamamoto@riken.jp (K.Y.); a_ogura@nagahama-i-bio.ac.jp (A.O.); 2 Kenya Research Station, Institute of Tropical Medicine, Nagasaki University, 1-12-4 Sakamoto, Nagasaki 852-8523, Japan; eawandera@gmail.com (E.A.W.); ichinose@nagasaki-u.ac.jp (Y.I.); 3 Department of Pediatric Infectious Diseases, Institute of Tropical Medicine, Nagasaki University, 1-12-4 Sakamoto, Nagasaki 852-8523, Japan; shah@nagasaki-u.ac.jp; 4 Department of Chemical Engineering, Fukuoka University, 8-19-1 Nanakuma, Jonan-ku, Fukuoka 814-0180, Japan; hirokazuseto@fukuoka-u.ac.jp; 5 Murata Manufacturing Co., Ltd., 1-10-1 Higashikotari, Nagaokakyo 617-8555, Japan; takashi_kondo@murata.com

**Keywords:** aerosol, bioaerosol, next generation sequencing, metal mesh devices (MMDs), particulate matter, pathogenic bacteria

## Abstract

Metal mesh devices (MMDs) are novel materials that enable the precise separation of particles by size. Structurally, MMDs consist of a periodic arrangement of square apertures of characteristic shapes and sizes on a thin nickel membrane. The present study describes the separation of aerosol particles using palm-top-size collection devices equipped with three types of MMDs differing in pore size. Aerosols were collected at a farm located in the suburbs of Nairobi, Kenya; aerosol particles were isolated, and pathogenic bacteria were identified in this microflora by next-generation sequencing analysis. The composition of the microflora in aerosol particles was found to depend on particle size. Gene fragments were obtained from the collected aerosols by PCR using primers specific for the genus *Mycobacterium*. This analysis showed that *Mycobacterium obuense*, a non-tuberculous species of mycobacteria that causes lung diseases, was present in these aerosols. These findings showed that application of this MMD analytical protocol to aerosol particles can facilitate the investigation of airborne pathogenic bacteria.

## 1. Introduction

The rapid separation and detection of aerosol particles in the field is essential for the assessment of environmental pollutants [1,2]. Aerosols, also called particulate matter (PM), have been shown to cause lung diseases when inhaled by humans, leading to their legal regulation in many countries. Of particular concern are particles <10 μm in diameter (PM10), which can invade the lungs, and particles <2.5 μm in diameter (PM2.5), which can invade deep lung tissue and the subepithelial environment. These fine particles have been shown to adversely affect health through oxidative stress and are associated with risks of allergies, asthma, cardiovascular diseases, and silicosis/pulmonary fibrosis [3,4,5,6]. In addition, since soil, water, sewage, and animal waste are major sources of aerosols, bacteria and viruses in these materials can bind to various kinds of aerosol particles, which contributes significantly to their widespread distribution. Among these aerosol particles, PM2.5 is expected to be associated with the transmission of respiratory infections because of its small size, which allows it to penetrate deeply into the respiratory system. Indeed, PM2.5 concentration is a factor related to bacterial community structure in air [7,8].

Several studies have assessed bioaerosols in natural environments. For example, pathogens detected in dust obtained from arid climates in Africa have been associated with local infections and allergies [9,10,11]. The effects of environmental bioaerosols have also been evaluated. For example, workers in the waste recycling industry, who are often exposed to very high levels of microorganisms, have high respiratory symptoms and airway inflammation [1]. In addition, occupational and non-occupational exposure to legionella bacteria in bioaerosols has been found to cause legionellosis [12,13,14]. These legionella bacteria have been found in many aquatic environments, including natural and artificial water systems, such as bathrooms, cooling systems, and water misting systems, leading to legionellosis outbreaks. Epidemiological studies have assessed the transmission by aerosols of infectious diseases, including Kawasaki disease [15] and SARS [16,17]. In addition, the recent worldwide outbreak of SARS-CoV-2 shows that airborne infections caused by bioaerosol inhalation can limit human activities and pose a risk of serious economic damage to society [18,19,20].

Aerosols are frequently collected by filtration because of the convenience of collecting samples using relatively little equipment [9,10,11,21]. Our research group has developed novel membrane filters, called metal mesh devices (MMDs), to fractionate PM for qualitative and quantitative evaluation. MMDs consist of a periodic structure of square apertures of characteristic size, arranged on a thin nickel membrane. In contrast to common synthetic resin membrane filters, MMDs have uniform pores and thus exhibit superior size-exclusion separation [22]. In addition, MMDs can only transmit electromagnetic waves in the frequency range determined by their periodic structure [23]. Because this optical property is dependent on the amount of material trapped on the MMDs, these membranes can be employed as label-free optical sensors. MMDs have been shown to easily separate and detect proteins, DNA, and living cells, as well as to fractionate and evaluate PMs both qualitatively and quantitatively [24,25]. 

The present study describes the development of a method to evaluate airborne bacteria, combining precise fractionation using MMDs and metagenomic analysis using next-generation sequencing (NGS). The aim of this study is to establish a method for evaluating airborne bacterial flora, which are difficult to evaluate using conventional methods, and to assess the associations between PMs and airborne bacteria for use in infection prevention. An analytical protocol that included a simple collection method for bioaerosols was therefore established. 

Tuberculosis is a serious health problem in developing countries in Africa [26]. Because tuberculosis is an airborne infectious disease, analysis of bioaerosols may lead to a method to prevent infection. Thus, bioaerosols were collected and analyzed using MMDs during the dry season, when the climate is stable, from a farm in Githunguri District, a suburb of Nairobi County, Kenya. The farm was considered a suitable site for a first trial of this technology because a relatively diverse range of plants and livestock are cultivated at the farm, suggesting that the bacterial flora would be diverse. Bacterial DNA was extracted from these collected aerosol particles and subjected to metagenomic analysis to evaluate the bacterial composition in the air. The results of these analyses demonstrated that MMDs could fractionate aerosol particles by size, enabling the subsequent analysis of microflora and the detection of specific pathogens in the collected samples.

## 2. Materials and Methods

### 2.1. Preparation of the MMD Filter Units

**Figure 1:**
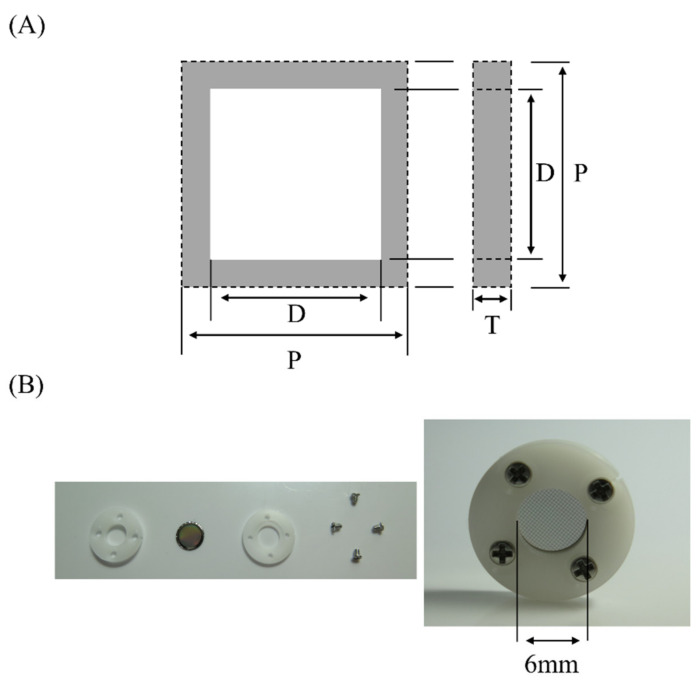
Image of the metal mesh device (MMD). (**A**) Dimensions of a single unit cell representative of the three types of MMD. D: aperture diameter of MMD; P: period of MMD; T: thickness of MMD. (**B**) An MMD packaged in polyoxymethylene (POM).

Figure 1A and Table 1 show the dimensions of single unit cells of three types of MMDs. The MMDs were made from nickel and were manufactured using the electroforming method. The MMDs were washed three times with 99.5% ethanol and pure water. After complete drying, the MMDs were irradiated with excimer light (wavelength: 172 nm) generated by an Excimer Photon Source Power Supply (Ushio Inc., Tokyo, Japan) filled with N_2_ gas. Each MMD was fixed in a polyacetal cover with an opening 6 mm in diameter (Figure 1B).

### 2.2. Collection of Aerosol Particles

**Figure 2:**
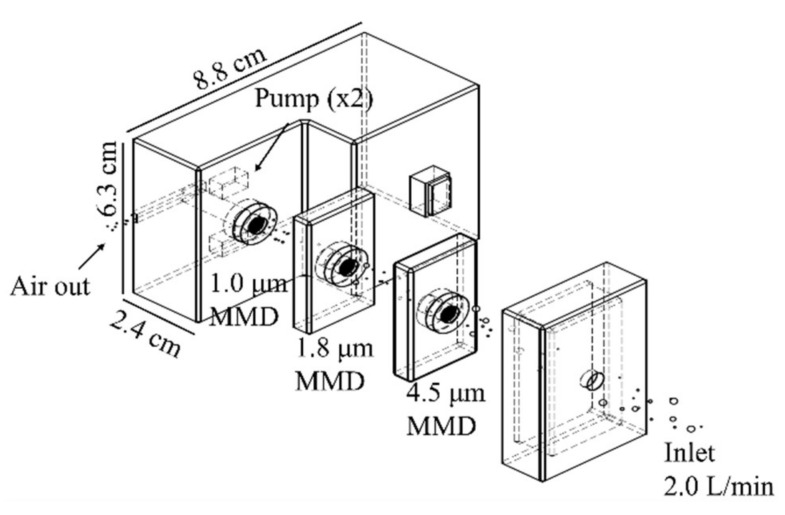
Schematic diagram showing the setup of the suction devices using stacked MMD sensors to capture aerosol particles according to size. A low-flow-rate air suction device used 4.5 µm, 1.8 µm, and 1.0 µm MMDs for collection of PM10, PM2.5, and PM1.0, respectively.

A new type of portable air suction device was fabricated to capture aerosols from the air (Figure 2). The two small air suction elements of this device are battery powered and can draw 2 L/min (static pressure) of air from the air suction port. A stack of three types of MMDs, fixed in the order of 4.5 μm, 1.8 μm, and 1.0 μm MMDs, were placed at the suction port of this device, thus allowing air to pass through the MMDs. Suction was applied for about 12 h at an air suction volume of about 1440 L, and the aerosol particles in the air were separated by the three types of MMDs. 

Aerosols were collected on three separate days, starting on 14 September 2016, at a farm in Githunguri, a suburb of Nairobi, Kenya. With the approval of the farm owner, a suction device was placed on the roof of a cowshed in open air. Air was suctioned during the daytime on sunny days, with nine samples being collected. Temperature and humidity were recorded as meteorological conditions. Collected MMDs containing aerosols were stored under sterile containers at 4 °C.

### 2.3. Analysis of Particle Size Distribution

The surfaces of the MMDs were evaluated by scanning electron microscopy (SEM; S-3400N, Hitachi High-Tech Co., Tokyo, Japan). Four SEM images of each MMD were evaluated and the contour shapes of the particles were determined using ImageJ/Fiji image analysis software. Distribution graphs of particle size indicated by the long-axis diameter were generated using OriginPro (Version 2021b, OriginLab Corporation, Northampton, MA, USA).

### 2.4. Particle Counting by IR Transmittance Measurements

The transmittance IR spectra of the 1.8 μm MMD were acquired using Fourier transform infrared spectroscopy (FT/IR-6600, JASCO International Co., Ltd., Tokyo, Japan). The wavenumber resolution, cumulative number, and diameter values were set to 2 cm^−1^ (0.06 THz), eight measurements, and 6 mm, respectively. A previously described method was used to estimate the number of aerosol particles from the frequency shift [23,24]. The frequency shift (−Δƒ) was calculated from the difference in the dip of the transmittance peak before and after the capture of aerosol particles and was corrected by subtracting the frequency shift of the MMD not exposed to aerosol particles. The calibration curve for obtaining the number of particles for these frequency shift values was prepared as follows. The aerosol was collected in Nagahama, Japan and the number of particles on the MMD was counted using a phase contrast microscope (CX31, Olympus Co., Tokyo, Japan) at a magnification of 200×. The average number of particles present in an area of 500 μm × 500 μm (*n* = 3) was calculated to obtain an estimate of the surface area of the MMD (28.3 mm^2^). The number of aerosol particles collected in Kenya was estimated using a calibration curve obtained using 1.8 μm MMDs.

### 2.5. Extraction of DNA from Aerosol Particles

DNA was extracted from the collected aerosols using NucleoSpin^®^ Soil kits (Takara Bio Inc., Shiga, Japan). Briefly, MMDs containing aerosols were removed from the polyacetal containers and transferred to NucleoSpin^®^ soil bead tubes containing ceramic beads. DNA was subsequently extracted using the protocol described by the manufacturer, with each sample yielding 40 μL of the final DNA extraction solution.

### 2.6. Preparation of 16S rDNA Samples

The V3–V4 region of 16S rDNA in the DNA samples extracted from the aerosol particles was PCR amplified on a PCR thermal cycler SP (Model. TP400, Takara Bio, Kusatsu, Japan), using the primers 341F/R805 (Table 2) [27,28]. Each 50 µL reaction mixture contained 12 µL of DNA template, Tks Gflex™ DNA polymerase (Code. R060A, Takara Bio, Kusatsu, Japan), and 1 µM of each primer. The amplification protocol consisted of 40 cycles of denaturation at 94 °C for 30 s, annealing at 50 °C for 30 s, and extension at 72 °C for 30 s. Indexed NGS adapters were attached to each amplified DNA fragment isolated by the PCR products purification kit. Eight sets of forward primers (D501-08, Illumina Inc., San Diego, CA, USA) and two sets of reverse primers (D709-710, Illumina, San Diego, CA, USA) were used. A second PCR amplification was performed using the same protocol as above. Amplified DNA fragments were isolated from each solution using magnetic beads DNA isolation kits (AMPure XP, Beckman Coulter Inc., La Brea, CA, USA), with the quality of the 16S rDNA fragments determined using a capillary electrophoresis device (MultiNA, Shimadzu Co., Kyoto, Japan). DNA concentrations were measured using Qubit dsDNA HS assay kits (Thermo Fisher Scientific Inc., Waltham, MA, USA) and a Qubit fluorometer (Thermo Fisher Scientific, Waltham, MA, USA).

### 2.7. Metagenomic Analysis Based on 16S rDNA Sequence Data

The sequences of the obtained 16S rDNA samples were determined by NGS sequencing (Miseq, Illumina, San Diego, CA, USA), with the 16S rDNA sequences analyzed using the QIIME2 (Quantitative Insights Into Microbial Ecology; 2020.8) microbiome bioinformatics platform 15 [29,30]. To eliminate noisy reads, DADA2, a plugin for QIIME2, was used to eliminate sequence data with a quality score of <25 for both forward and reverse reads, with the remaining 75% of the data was used for further analysis. The clustering sequences from these data were treated as operational taxonomic units (OTUs), allowing their classification into species or genera by taxonomic analysis with reference to the Greengenes database [31].

### 2.8. Detection of the Genus Mycobacterium

Multiplex PCR was performed using primers specific for the 16S rRNA gene of *Mycobacterium* spp. (Table 2) [32,33]. Each reaction solution contained template DNA, 1.25 U TaKaRa ExTaq HS, ExTaq buffer (Code. RR006A, Takara Bio, Kusatsu, Japan), and 1 µM of each primer. The amplification protocol consisted of 35 cycles of denaturation at 94 °C for 30 s, annealing at 62 °C for 1 min, and extension at 72 °C for 1 min. PCR products were electrophoresed on 2% agarose gels, which were stained with ethidium bromide. The amplified fragments were purified using AMPure XP magnetic beads DNA isolation kits, and each fragment was ligated to 50 ng pMD20 T-vector. Following vector transformation of *Escherichia coli* HST08, the subcloned vectors were obtained using *E. coli* HST08 Premium Competent (Takara Bio, Kusatsu, Japan) kits and extracted by the alkaline SDS method. The DNA inserts were subjected to DNA sequencing (Applied Biosystems 3130, Thermo Fisher Scientific, Waltham, MA, USA) using a method based on the big dye terminator cycle sequencing method and the M13 primers RV 3 (sense strand) and M4 (antisense strand), as appropriate.

## 3. Results

### 3.1. Microflora Analysis of Aerosol Particles on MMDs

**Figure 3:**
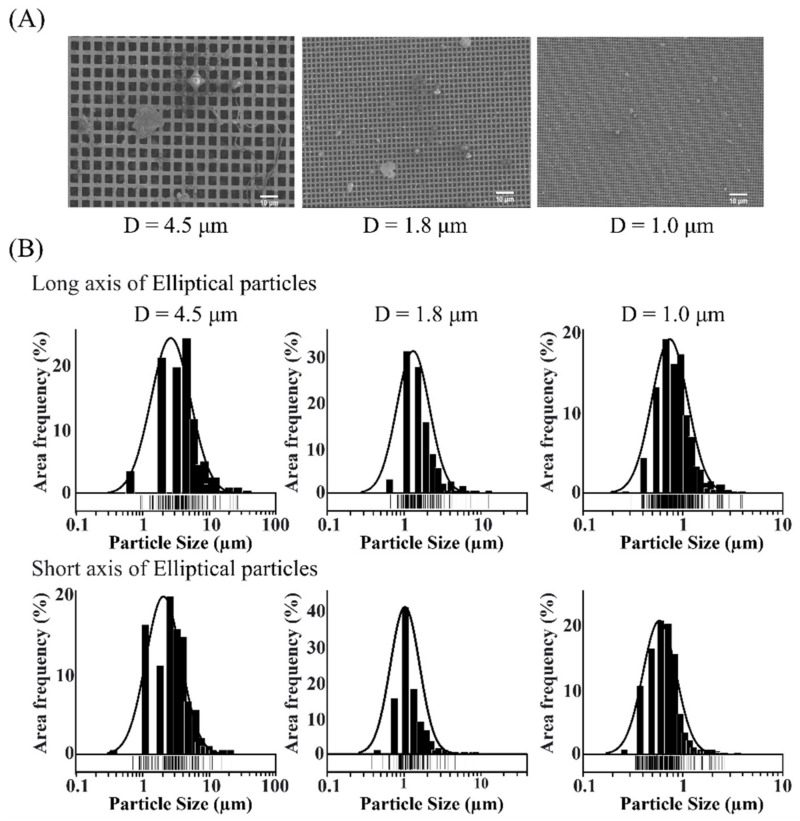
(**A**) SEM images of the surfaces of MMDs on which aerosol particles were collected with the low-flow-rate air suction device. Size bar, 50 μm. (**B**) Distribution of the sizes of aerosol particles captured on each pore size MMD.

Aerosol particles were collected by size using the air suction device (Figure 2), taking advantage of the fractionation capability of the stacked MMDs. The size-fractionated particles were evaluated by SEM (Figure 3A). The average long-axis sizes and numbers (n) of the particles recovered on the 4.5 μm, 1.8 μm, and 1.0 μm MMDs were 5.39 ± 4.9 μm (*n* = 201), 1.4 ± 1.4 μm (*n* = 202), and 0.97 ± 0.49 μm (*n* = 490), respectively (Figure 3B).

**Figure 4:**
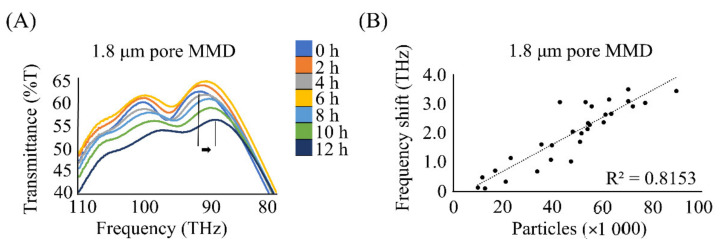
(**A**) Spectral changes over time for the 1.8 μm MMD, showing a shift of −3.15 THz after collecting aerosol particles for 12 h. (**B**) The regression line of particle number vs. frequency shift for particles captured by the 1.8 μm MMD (R^2^, 0.8153).

To estimate the number of particles from the peak shift in the IR transmission spectrum, a calibration curve was constructed using the relationship between the number of aerosol particles counted by the microscope and the transmission peak [23,24]. The 1.8 μm MMD had IR transmission characteristics that were approximately proportional to the number of aerosol particles collected (Figure 4). The regression line was used to estimate the number of aerosol particles collected in Kenya. The average value of the peak shifts of the collected MMDs in Kenya was 1.2 ± 0.3 THz, and the number of aerosol particles was estimated to be 3.1 ± 0.6 × 10^4^ particles/MMD.

**Figure 5:**
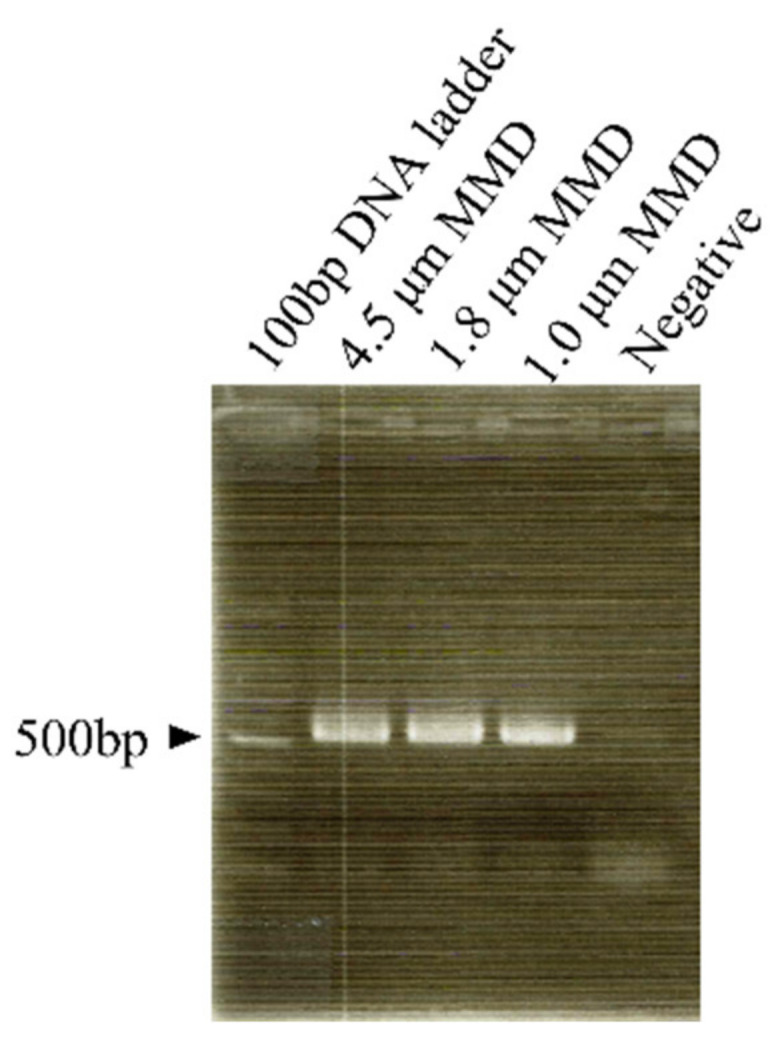
PCR results obtained universal primers for bacterial 16SrDNA amplification. Negative controls were subjected to the same procedures but without DNA extraction from MMD-captured aerosol particles.

Aerosol samples were collected by MMDs three times on three successive days. DNA was extracted from aerosols collected from three different MMDs, and PCR was performed using universal primers that amplify the V3–V4 region of bacterial 16S rDNA. The amount of DNA obtained after amplification from each MMD collection was confirmed to be approximately 40 ng by measurement using Qubit. Agarose electrophoresis showed that the molecular weight of the amplified DNA fragment corresponded to the length of the V3–V4 region (about 550 bp) (Figure 5). No DNA was amplified from samples extracted in the same procedure from not used MMDs as negative controls. 

The average numbers of families, genera, and species identified from each OTU are summarized in Table 3. The effective sequence information for each sample collected by the low-flow-rate suction device ranged from 95,000 to 115,000 reads, and the numbers of OTUs ranged from 87 to 139. The number of OTUs tended to decrease slightly as the pore sizes of the MMDs decreased.

**Figure 6:**
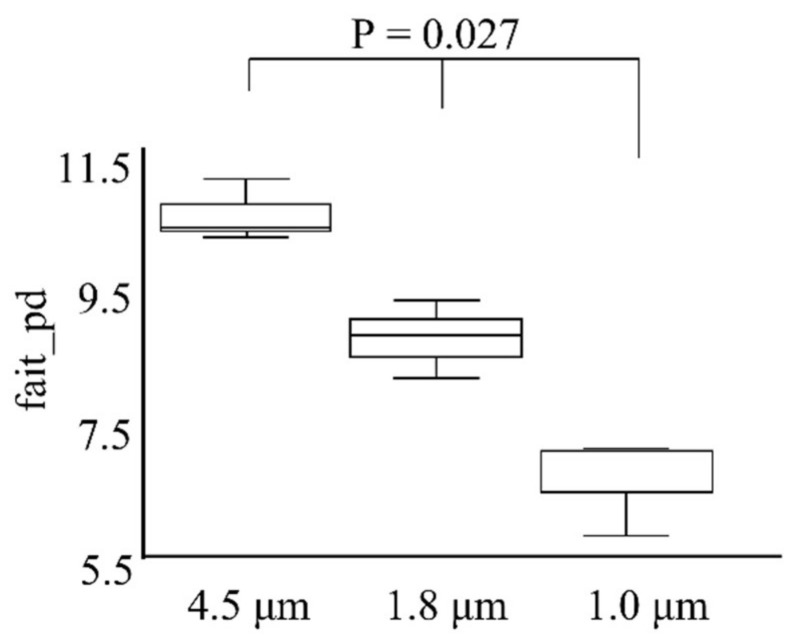
Boxplot of α-diversity indices, which reflect abundance and consistency, of microflora obtained from the MMDs at low- and high-flow rates. Boxes represent the interquartile range (IQR) between the first and third quartiles (25th and 75th percentiles, respectively), and the horizontal line inside each box represents the median. Whiskers represent the lowest and highest values within 1.5 times the IQR from the first and third quartiles, respectively. Diversity decreased significantly as MMD pore size decreased MMD. P-values were determined by Student’s *t*-tests.

Metagenomic data analysis was performed with QIIME2, with a comparison of α-diversity, a statistical index for phylogenetic diversity, shown in Figure 6. A larger index indicates the presence of more diverse bacteria. These results indicate that the α-diversity of microflora in aerosol particles tended to decrease as the pore size of the MMDs decreased.

The total number of OTUs obtained from the metagenome analysis of the airborne microflora collected by the MMDs was 198, with genus or species identified for 118 of these OTUs. The top 10 bacteria from the OTUs collected by each MMD are summarized in Table 4. The 34 bacterial species classified as pathogenic and their contents are summarized in Table 5. Six, seven, and seven pathogenic bacteria were collected by the 4.5 μm, 1.8 μm, and 1 μm MMDs, respectively.

### 3.2. Detection of Mycobacterium *spp.*

Tuberculosis, an airborne infectious disease caused by *Mycobacterium* spp., is highly prevalent in Kenya. This pathogen, however, was not detected in the above analyses of airborne bacteria. PCR-amplified fragments corresponding to those of *Mycobacterium* spp. were observed only in the flora of airborne bacteria collected by 4.5 μm pore-size MMDs. Sequence analysis showed that the 973 bp PCR product was 99% identical to a sequence of *Mycobacterium obuense*, a non-tuberculous *Mycobacterium* that causes lung disease.

## 4. Discussion

This study describes the development of a new sampling protocol in which atmospheric aerosols were size fractionated by small collectors equipped with MMDs, followed by genetic analysis to identify airborne microflora. Conventional technologies, such as high-volume filter samplers [34] and high-flow-rate impingers [35], require the collection of 1000–100,000 L or more of air to obtain biomass sufficient for downstream analysis. The volume of air collected by this technique was about 1500 L, a volume estimated to contain 10^2^–10^6^ bacteria [8]. Although this amount is smaller than the amount conventionally collected, the recovery of DNA from the MMD surface is excellent, and amplified fragments of the 16S ribosomal region were obtained by conventional PCR.

NGS analysis detected 87–139 OTUs in these samples. Stacking of three types of MMDs enabled the fractionation and analysis of bacterial composition and content from a smaller sample than the previously mentioned conventional techniques. The drawbacks of filtration include concerns about damage to the microorganisms caused by prolonged collection, the clogging of the pores by the particles, and the difficulty releasing particles following their attachment to the filter [11,36,37]. The pores of the MMDs described in this study have a large aperture ratio, preventing clogging of the pores until the surface is completely filled with collected particles [22,24]. Furthermore, because of the single thin-layer structure of the MMDs, the collected particles are exposed on their surfaces, making it easy to release the particles and to extract DNA from them by direct immersion in DNA extraction reagents.

Stacking MMDs with pore sizes of 4.5 μm, 1.8 μm, and 1 μm resulted in the fractionation of particles with average sizes of 5.39 ± 4.9 μm, 1.4 ± 1.4 μm, and 0.97 ± 0.49 μm, respectively, indicating the effectiveness of size fractionation by MMDs. The PCR amplification of bacterial 16S rDNA from these fractionated aerosol particles revealed the presence of intrinsic bacterial flora in each aerosol fraction.

The aerosol samples in this study were collected at a farm that grows a variety of crops and raises cattle, pigs, and chickens on a small scale. Livestock manure is composted and spread on the fields. Bacterial flora in the aerosols obtained from this environment were reported to have greater diversity than flora in aerosols obtained from areas in cities [38,39], a finding supported by the present results. Differences in floral diversity may have resulted from differences in propagation distance depending on particle size, in that smaller-sized particles may have collected airborne bacteria from a wider range of sources. 

*Pseudomonas*, on the other hand, was detected in all MMDs with different pore sizes. At the sampling sites, aerosols were assumed to originate from soil, livestock feed, and compost and were ejected into the atmosphere near the sampling sites. Considering the relationship between particle size and dispersal distance, it is assumed that aerosol particles fractionated in MMDs with larger pore sizes would have shorter dispersal distances and be collected from the vicinity of the collection site, while aerosols fractionated in MMDs with smaller pore sizes would have longer dispersal distances and be collected from a wider area. *Pseudomonas* is abundant in the environment and may be present in a wide range of aerosol particles, ranging from small to large.

The α-diversity indices suggested that the unique flora in aerosols depend on the size of the particles, with smaller particle sizes resulting in a greater difference. This difference could be due to factors such as the origin, dispersal distance, and differences in the protective effects of UV light and drying among different-sized particles [40], but analyses with larger numbers of samples are required.

In this study, we also assessed whether the sensitivity of detection of floating bacteria could be improved by primer selection. For example, to detect *Mycobacterium tuberculosis*, a highly prevalent species in Kenya, we utilized a tuberculosis-specific primer set (MYCGEN primers) [32] and attempted to detect this bacterium in the airborne bacteria collected on MMDs. Although 16S rDNA analysis did not detect *M. tuberculosis*, the MYCGEN primers detected *M. obuense* on the 4.5 μm pore-size MMDs. However, in the present study, fungal species and viruses were not targeted; therefore, further work will be required to diversify the application so that it can identify different microorganisms, including SARS-CoV-2 viruses.

This study was able to identify pathogenic bacteria in plants, including *Pseudomonas syringae*, *Clostridium disporicum*, and *Rhodococcus fascians*, as well as bacteria associated with infectious diseases in animals, including *Corynebacterium pilosum*, which induces cystitis and pyelonephritis in cattle, *Enterococcus cecorum*, which causes bacterial infections in pigs, calves, and other species, *Streptococcus equi*, which induces streptococcal mastitis in cattle and acute septicemia in poultry, and *Staphylococcus saprophyticus*, which induces cystitis [41]. Analyses of the bacterial flora in dust from arid climates in Africa have identified several genera and species of pathogenic bacteria observed in this study [9,10,11,42]. Although most of the pathogens identified in the study were opportunistic, exposure to dust on farms with livestock has been shown to pose some risks to human health [42]. 

Bioaerosols have a wide particle-size distribution, ranging from 0.01 μm (viruses) to 100 μm (pollen). Bioaerosols are often found mixed with other matter, such as mineral dust or sea salt. Because many of these particles adsorb to each other to form composite particles, bioaerosols can change in size depending on their source and time course. Since the pore size of the MMDs can be strictly regulated, the size fractionation performance of the MMDs is useful in elucidating bioaerosol characteristics and particle size. One limitation is that the lower pore-size limit of current MMDs is 1 μm, which is too large to capture viruses floating alone. However, most viruses are likely to be adsorbed on the carrier particles [43], and it is thought that this limitation can be compensated for when the MMD is combined with a highly sensitive detection method. 

In the future, MMDs are expected to establish detection protocols for pathogenic bacteria and viruses, especially coronaviruses, which will make them useful in hospitals and workplaces as a tool for judging the effectiveness of infection prevention and control. The developed aerosol collector is compact and quiet, making it also suitable for indoor aerosol collection. This feature is attributed to the MMD’s large aperture ratio and low pressure resistance. We plan to perform further testing of the MMDs in a hospital facility specializing in tuberculosis. The aerosol collector has advantages in that it can be used in hospital rooms without stressing patients or hospital staff.

## 5. Conclusions

The present study describes the utilization of new MMDs to analyze bacteria in air samples at a farm in the suburbs of Nairobi, Kenya. MMDs are substrate materials consisting of thin membranes with a periodic structure on smooth surfaces that can be used to detect and separate particles. Aerosol particles, including the damaging environmental pollutant PM2.5, were separated based on size and captured on MMD surfaces using a small air pump. Bacterial genomic DNA could be extracted at high yield from aerosol particles on MMDs, with NGS analysis of bacterial 16S rDNA sequences showing the bacterial constituents of these air samples. These results provided information on the bacterial biota in the local environment, including the identification of pathogenic bacteria. MMDs can be considered a simple monitoring device for the detection and quantification of airborne pathogens.

## Figures and Tables

**Table 1:** Properties of the MMDs used in this study.

Aperture Diameter (D)	Thickness (T)	Period (P)
1.0 μm	0.8 μm	1.4 μm
1.8 μm	1.0 μm	2.6 μm
4.5 μm	1.0 μm	6.4 μm

**Table 2:** Primers used in this study.

Name	Primer Sequence (5′–3′)
341F	TCGTCGGCAGCGTCAGATGTGTATAAGAGACAGCCTACGGGNGGCWGCAG
R805	GTCTCGTGGGCTCGGAGATGTGTATAAGAGACAGGACTACHVGGGTATCTAATCC
MYCGEN-F	AGAGTTTGATCCTGGCTCAG
MYCGEN-R	TGCACACAGGCCACAAGGGA

**Table 3:** Metagenome analysis of 16S rDNA extracted from aerosol particles collected with the air suction devices.

MMD Size	Reads	OTUs	Family	Genus	Species
4.5 μm	104,884 ± 6138	121 ± 18	90 ± 16	50 ± 12	26 ± 7
1.8 μm	105,624 ± 9921	104 ± 11	83 ± 8	53 ± 7	23 ± 5
1.0 μm	112,534 ± 2784	92 ± 6	71 ± 4	53 ± 4	25 ± 2

**Table 4:** The top 10 bacterial isolates identified from air samples collected by MMDs installed in the air suction device. Content rates are mean values (*n* = 3) of each OTU read number for the total NGS reads.

Rank	Bacteria	Content Rate (%)
4.5 μm MMDs		
1	*Pseudomonas* spp.	48.896
2	*Gammaproteobacteria*	17.592
3	*Streptophyta*	5.589
4	*Betaproteobacteria*	4.340
5	*Enterobacteriaceae*	4.334
6	*Actinomycetales*	2.453
7	*Propionibacterium* spp.	1.978
8	*Staphylococcus saprophyticus*	1.500
9	*Corynebacterium* spp.	1.430
10	*Enhydrobacter aerosaccus*	0.582
1.8 μm MMDs		
1	*Pseudomonas* spp.	49.908
2	*Gammaproteobacteria*	22.097
3	*Enterobacteriaceae*	10.825
4	*Nostocales*	1.424
5	*Enhydrobacter aerosaccus*	1.022
6	*Betaproteobacteria*	0.944
7	*Pseudomonas syringae*	0.899
8	*Acinetobacter* spp.	0.863
9	*Actinomycetales*	0.804
10	*Staphylococcus saprophyticus*	0.564
1.0 μm MMDs		
1	*Pseudomonas* spp.	55.843
2	*Gammaproteobacteria*	24.476
3	*Enterobacteriaceae*	5.016
4	*Enhydrobacter aerosaccus*	1.371
5	*Staphylococcus saprophyticus*	1.012
6	*Pseudomonas syringae*	0.963
7	*Actinomycetales*	0.838
8	*Betaproteobacteria*	0.787
9	*Acinetobacter* spp.	0.533
10	*Propionibacterium* spp.	0.525

**Table 5:** Pathogenic bacteria identified from air samples collected by MMDs installed in the air suction devices. Content rates are mean values (*n* = 3) of each OTU read number for the total NGS reads.

		Content Rate (%)		
NO	Bacteria	4.5 μm	1.8 μm	1.0 μm
1	*Pseudomonas* spp.	48.896	49.908	55.843
2	*Staphylococcus saprophyticus*	1.5	0.564	1.012
3	*Propionibacterium* spp.	1.978	0.553	0.525
4	*Pseudomonas syringae*	0.553	0.899	0.963
5	*Corynebacterium* spp.	1.43	0.308	0.273
6	*Acinetobacter* spp.	0.393	0.863	0.533
7	*Streptococcus* spp.	0.337	0.394	0.067
8	*Elizabethkingia* spp.	0.128	0.059	0.106
9	*Corynebacterium pilosum*	0.006	0.137	0.074
10	*Peptoniphilus* spp.	0	0.16	0
11	*Anaerococcus* spp.	0.145	0	0
12	*Streptococcus equi*	0.058	0.087	0
13	*Bordetella ansorpii*	0	0	0.126
14	*Brevundimonas vesicularis*	0.091	0.03	0
15	*Bacillus* spp.	0	0	0.116
16	*Roseomonas* spp.	0.112	0	0.004
17	*Corynebacterium simulans*	0	0.111	0
18	*Haematobacter massiliensis*	0	0.106	0
19	*Finegoldia* spp.	0	0.103	0
20	*Clostridium paraputrificum*	0	0	0.097
21	*Enterococcus cecorum*	0.055	0.035	0
22	*Helicobacter* spp.	0	0	0.089
23	*Propionibacterium granulosum*	0.072	0.01	0
24	*Kocuria kristinae*	0	0.071	0
25	*Rhodococcus fascians*	0.063	0	0
26	*Rhodococcus* spp.	0	0.061	0
27	*Aeromonas* spp.	0	0	0.044
28	*Bacteroides* spp.	0.043	0	0
29	*Brevibacterium casei*	0	0	0.035
30	*Brevundimonas* spp.	0	0.034	0
31	*Helcobacillus massiliensis*	0	0	0.022
32	*Methanobrevibacter* spp.	0.015	0	0
33	*Bordetella* spp.	0.003	0	0
34	*Rothia* spp.	0.002	0	0

## Data Availability

All relevant data can be found in the paper.
